# Combined SNPs sequencing and allele specific proteomics capture reveal functional causality underpinning the 2p25 prostate cancer susceptibility locus

**DOI:** 10.21203/rs.3.rs-3943095/v1

**Published:** 2024-04-04

**Authors:** Gong-Hong Wei, Dandan Dong, Peng Zhang, Mengqi Liu, Yu Wei, Zixian Wang, Wenjie Xu, Qixiang Zhang, Yao Zhu, Qin Zhang, Xiayun Yang, Jing Zhu, Liang Wang

**Affiliations:** Fudan University Shanghai Cancer Center & MOE Key Laboratory of Metabolism and Molecular Medicine and Department of Biochemistry and Molecular Biology of School Basic Medical Sciences, Shanghai Medi; Shanghai Medical College of Fudan University; Shanghai Medical College of Fudan University; Shanghai Medical College of Fudan University; Fudan Unversity Shanghai Cancer Center; Shanghai Medical College of Fudan University; Shanghai Medical College of Fudan University; Shanghai Medical College of Fudan University; Fudan University Shanghai Cancer Center; University of Oulu; Biocenter Oulu, University of Oulu; Harbin Medical University; Moffitt Cancer Center

**Keywords:** Prostate cancer, SNPs-seq, rs4519489/2p25 locus, proteomics, NOL10, USF1

## Abstract

Genome wide association studies (GWASs) have identified numerous risk loci associated with prostate cancer, yet unraveling their functional significance remains elusive. Leveraging our high-throughput SNPs-seq method, we pinpointed rs4519489 within the multi-ancestry GWAS-discovered 2p25 locus as a potential functional SNP due to its significant allelic differences in protein binding. Here, we conduct a comprehensive analysis of rs4519489 and its associated gene, NOL10, employing diverse cohort data and experimental models. Clinical findings reveal a synergistic effect between rs4519489 genotype and NOL10 expression on prostate cancer prognosis and severity. Through unbiased proteomics screening, we reveal that the risk allele A of rs4519489 exhibits enhanced binding to USF1, a novel oncogenic transcription factor (TF) implicated in prostate cancer progression and prognosis, resulting in elevated NOL10 expression. Furthermore, we elucidate that NOL10 regulates cell cycle pathways, fostering prostate cancer progression. The concurrent expression of NOL10 and USF1 correlates with aggressive prostate cancer characteristics and poorer prognosis. Collectively, our study offers a robust strategy for functional SNP screening and TF identification through high-throughput SNPs-seq and unbiased proteomics, highlighting the rs4519489-USF1-NOL10 regulatory axis as a promising biomarker or therapeutic target for clinical diagnosis and treatment of prostate cancer.

## Introduction

Prostate cancer is the second most common cancer and the fifth leading cause of cancer-related mortality among men worldwide, with around 1.4 million new cases and 400,000 deaths annually^1^. The disease’s incidence and mortality rates vary significantly by region, with the highest incidence observed in Northern and Western Europe and the lowest in Asia^1^. Notably, mortality rates have decreased in regions like Northern America, Oceania, and Northern and Western Europe. However, recent years have seen an increase in both incidence and mortality rates in Asia, Central and Eastern Europe, and sub-Saharan Africa^1, 2^. This rise is likely due to improved awareness, widespread use of prostate-specific antigen (PSA) testing, alongside rising incidence trends and challenges in accessing effective treatment options^1–7^.

Prostate cancer development is influenced by a complex interplay of factors, including age, familial history, genetic background, germline mutations, and lifestyle/environmental factors like smoking, obesity, and diet^1, 8^. Oncogenic pathways in prostate cancer features a range of genetic alterations, including somatic mutations in crucial genes (SPOP, FOXA1, TP53, AR, RB1), PTEN deletions, MYC amplifications, and gene fusions like TMPRSS2-ERG^1, 8^. Our previous research highlighted the intricate interplay between the somatic TMPRSS2-ERG fusion and the 17q12/HNF1B locus^9^, underlining their significant roles in prostate cancer risk and progression.

Large-scale twin studies and epidemiological evidence have revealed a significant genetic component to prostate cancer, estimating its heritability at 57%^10, 11^. The introduction of genome-wide association studies (GWASs) has fundamentally enhanced our understanding of the genetics underlying prostate cancer^10, 12^, identifying over 450 susceptibility variants since the first GWAS in 2005, as documented in the NHGRI-EBI GWAS Catalog^10, 12–15^. Post-GWAS research is now focused on exploring the biological mechanisms underpinning these susceptibility loci, uncovering risk loci that affect crucial processes in prostate tumorigenesis, such as cell cycle regulation, DNA repair, inflammation, and metabolism^16, 17^. A key challenge remains from association studies to functional investigations, with a keen emphasis on their potential clinical implications and applications^17–21^.

Recent progress in high-throughput screening approaches has significantly enhanced the annotation of functional single nucleotide polymorphisms (fSNPs), connecting GWAS outcomes to disease mechanisms. Techniques such as the massively parallel reporter assay (MPRA) allow for the examination of thousands of sequences for potential transcriptional activation, enabling detailed analysis of transcriptional regulatory elements with genetic variations^22, 23^. The self-transcribing active regulatory region sequencing (STARR-seq) method quantitatively evaluates enhancer activity across millions of sequences harboring regulatory SNPs^24, 25^. We and others have employed CRISPR interference (CRISPRi) to identify regulatory elements and their target genes, clarifying the role of noncoding genetic variation in prostate cancer^26, 27^. Pooled chromatin immunoprecipitation sequencing (pooled ChlP-seq) links genetic variants in transcription factor binding to disease risk^28^. Additionally, our team previously developed an innovative high-throughput technique called single-nucleotide polymorphisms sequencing (SNPs-seq)^29^, together with the type IIS enzymatic restriction approach developed by Li and colleagues^30^, enables the identification of fSNPs influencing allele-specific regulatory protein binding and thereby further bridging the gap between genetic variants and their functional impact on diseases.

Our SNPs-seq method capitalizes on the selective retention of protein-bound DNA oligonucleotides in a protein purification column, followed by massively quantitative sequencing. Using it for a broad analysis of fSNPs at prostate cancer risk loci, we identified numerous candidate fSNPs^29^. Notably, rs4519489 at the 2p25 locus, located in an intron of the nucleolar protein 10 (NOL10) gene, showed significant allelic variation in protein binding. Further underlining its significance, several large-scale GWASs have discovered the 2p25 locus as significant for prostate cancer susceptibility and severity with two lead SNPs, rs9287719 and rs1990613^14, 15, 31, 32^ showing strong linkage disequilibrium with rs4519489 and thus emphasizing its functional role in prostate cancer causality. Herein we conducted a thorough functional analysis of rs4519489 and its eQTL target gene, NOL10. We discovered that the transcription factor USF1 plays a crucial role in modulating NOL10 expression through rs4519489, using an unbiased proteomics approach. Our research further investigates the impact of NOL10 and USF1 on prostate cancer predisposition and progression.

## Results

### Identification of functionally critical variants and eQTL genes underlying GWAS loci of prostate cancer

**Figure 1:**
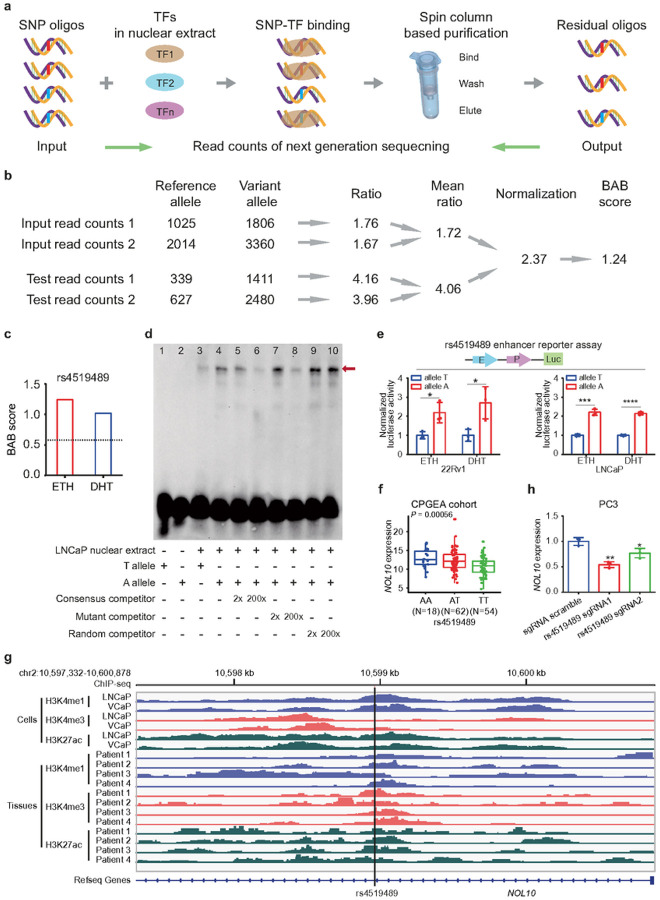
Analysis of allele-specific protein binding at rs4519489/2p25 locus and its association with NOL10 expression. **a**Identification of significant SNPs with allele-dependent protein binding differences using SNPs-seq. **b**Methodology for calculating the biased allelic binding (BAB) score. **c** Enhanced binding preference of the A allele over the T allele of rs4519489 with ETH or DHT treatment, as evidenced by SNPs-seq results. **d**Electrophoresis mobility shift assay (EMSA) demonstrating higher binding affinity of the A allele (lane 4) compared to the T allele (lane 3) of rs4519489. Binding is displaced by a 200× consensus competitor (lane 6) and diminished by a 200× mutant competitor (lane 8), but unaffected by a 200× random competitor (lane 10). **e**Luciferase reporter assay indicating increased enhancer activity (571 bp DNA segment, chr2: 10,598,681–10,599,251, Human GRCh38) with the A allele of rs4519489 compared to the T allele in 22Rv1 and LNCaP cells under ETH or DHT treatment (E, enhancer; P, promoter; Luc, luciferase). **f**Association of the risk allele A at rs4519489 with elevated NOL10 expression in the CPGEA cohort. **g**ChIP-seq data revealing histone modification enrichment (H3K4me1, H3K4me3, and H3K27ac) at the 4519489/2p25 locus in prostate cancer cell lines (LNCaP and VCaP) and prostate tissues (normal and cancerous). **h**Investigation of sgRNA-targeted CRISPRi effects on NOL10 expression at the rs451948 region. *P<0.05, **P<0.01, via two-tailed student’s T test.

Identifying functionally critical variants and their linked expression quantitative trait loci (eQTL) genes within GWAS loci is crucial for unraveling the genetic complexity of prostate cancer. This requires integrating various techniques, including high-throughput screening, allele-specific assays, and analyses correlating genotypes with phenotypes. Our recent work leverages our innovative SNPs-seq method to study 374 prostate cancer risk loci, examining allelic differences in protein binding^29^. Results showed notable allele-dependent binding variations (Fig. 1a); specifically, the A allele of rs4519489 had stronger protein binding than the T allele, with significant biased allelic binding (BAB) scores in different samples (Fig. 1b, c). Critically, the A allele of rs4519489 strongly correlates with the two major GWAS risk SNPs, namely the C allele of rs9287719 (R^2^ = 0.67, D’=0.82)^31, 32^ and the T allele of rs1990613 (R^2^ = 0.8, D’=0.98)^14, 15^, underscoring its potential functional importance in prostate cancer genetics.

To confirm the distinct protein binding affinities of rs4519489 T and A alleles, we performed an electrophoretic mobility shift assay (EMSA). The A allele showed stronger binding to nuclear protein binding in LNCaP cells than the T allele. Binding for the A allele was significantly reduced by a consensus competitor but unaffected by mutant or random competitors (Fig. 1d). Additionally, we investigated transcriptional regulation differences using an allele-specific enhancer reporter assay. A 571 bp DNA segment containing either allele of rs4519489 was cloned into luciferase reporter vectors. The A allele produced a higher luciferase activity than the T allele in both 22Rv1 and LNCaP cells, with or without dihydrotestosterone (DHT) treatment, indicating its stronger transcriptional activation potential (Fig. 1e).

Subsequently, to assess if the rs4519489 SNP genotype correlates with gene expression nearby, we conducted an eQTL analysis using the Chinese Prostate Cancer Genome and Epigenome Atlas (CPGEA) cohort^33^. The analysis linked the aggressive prostate cancer-associated A allele of rs4519489 with higher NOL10 mRNA expression (Fig. 1f; **Supplementary Dataset 1**), suggesting an involvement of NOL10 aggressive prostate cancer predisposition.

Further, to examine the potential enhancer functionality of the rs4519489 region, we performed chromatin immunoprecipitation sequencing (ChIP-seq) experiments for epigenetic markers in various cell lines and clinical specimens. The results, shown in the Integrative Genomics Viewer, indicated active epigenetic marker enrichment (H3K27ac, H3K4me1, H3K4me3) at the rs4519489 locus (Fig. 1g), hinting at regulatory elements presence. Expanding ChIP-seq to include histone modifications in both normal and tumor prostate tissues from the CPGEA cohort^33^ confirmed enhancer/promoter activity at rs4519489 (Fig. 1g), reinforcing its functional gene regulatory role.

To explore if the rs4519489 region acts as an enhancer affecting NOL10 expression, we utilized CRISPR interference (CRISPRi) in PC3 cells. We first established a cell line with stable dCas9 expression, then designed and integrated two sgRNAs targeting the rs4519489 enhancer region into a humanized pgRNA vector (including an sgRNA targeting HPRT as a positive control). After infecting these dCas9-expressing cells with a lentivirus carrying the sgRNA plasmids, RT-qPCR analysis showed a notable decrease in NOL10 mRNA levels upon targeting the rs4519489 enhancer (Fig. 1h), indicating its role in modulating NOL10 expression.

In summary, our high-throughput SNPs-seq screening identified rs4519489 as a functional causal SNP closely linked with key GWAS lead SNPs at the 2p25 prostate cancer susceptibility locus. Genotype-expression analysis revealed NOL10 as the eQTL gene for rs4519489, indicating the rs4519489/2p25 region likely functions as an enhancer modulating NOL10 expression.

### NOL10 upregulation and rs4519489 eQTL correlate with prostate cancer severity

**Figure 2:**
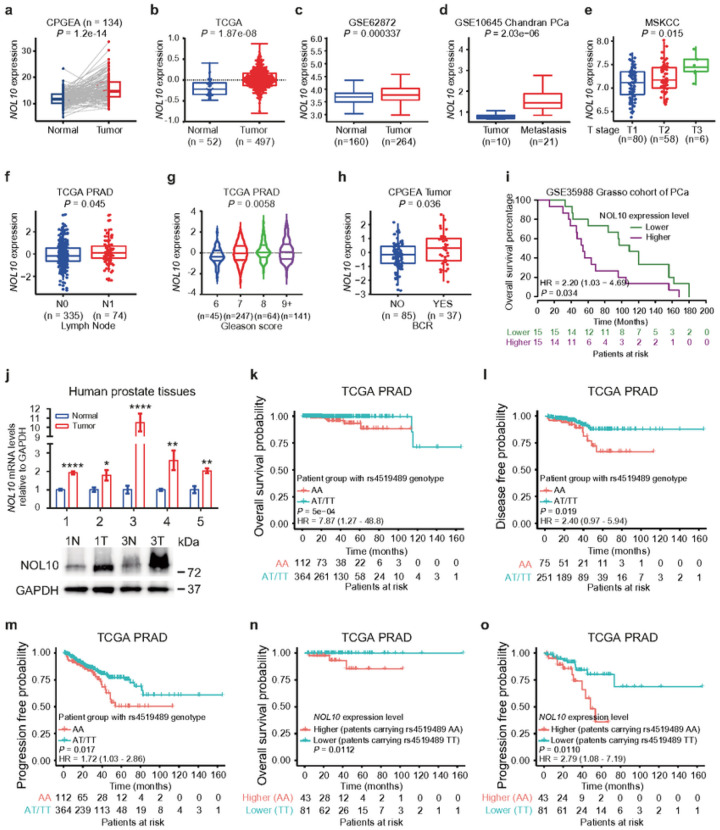
Correlation of NOL10 expression, rs4519489 genotype with prostate cancer risk and severity. **a-c**Elevated NOL10 expression in prostate adenocarcinoma compared to normal prostate glands across CPGEA, TCGA, and GSE62872 cohorts. **d-h** Association of high NOL10 expression with various clinical features: increased tumor metastasis **(d**, n=31), higher tumor stage **(e**, n=144), lymph node metastasis **(f**, n=409), higher Gleason score **(g**, n=497), and biochemical recurrence (BCR) **(h**, n=122) in prostate cancer patients. **i** Higher NOL10 levels correlating with reduced overall survival in the GSE35988 cohort. **j** Analysis of NOL10 expression in prostate cancer versus paracancerous tissues using qRT-PCR and western blot, with GAPDH as a loading control. **k-m**Prostate cancer patients with rs4519489 AA genotype in the TCGA cohort showing lower overall survival, disease-free, and progression-free probability. **n-o** Worse overall survival and progression-free probability in TCGA cohort patients with rs4519489 AA genotype and higher NOL10 expression tumors. *P<0.05, **P<0.01, ***P<0.001, ****P<0.0001; analyzed using two-way ANOVA.

To ascertain the functional significance of NOL10 in the clinical settings, we initially analyzed the CPGEA data^33^, and revealed significant upregulation of NOL10 mRNA in prostate cancer tumors compared to normal tissues (Fig. 2a). This finding was supported by further analysis of data from TCGA PRAD^34^, Health Study Prostate Tumor Cohort^35, 36^, and another Chinese prostate cancer dataset^37^, all of which consistently indicated higher NOL10 expression in tumor tissues (Fig. 2b, c and **Supplementary Fig. S1a**). Independent validation using samples from the Fudan University Shanghai Cancer Center (FUSCC) cohort^38^, through RT-qPCR and Western Blot, confirmed significant overexpression of NOL10 in prostate tumor tissues (Fig. 2j). Additionally, analysis of the GSE10645 dataset^39^ showed a notable association between NOL10 expression and metastatic progression in prostate cancer patients (Fig. 2d), underscoring NOL10 upregulation and its rs4519489 eQTL correlation with the severity of prostate cancer..

Our in-depth analysis of clinical features in prostate cancer patients revealed that elevated NOL10 expression correlates significantly with more advanced tumor stages^34, 40^ (Fig. 2e and **Supplementary Fig. S1b**), lymph node metastasis^34^ (Fig. 2f), higher Gleason scores^34^ (Fig. 2g), and increased biochemical recurrence rates^33^ (Fig. 2h). Moreover, survival analysis utilizing the Grasso dataset^41^ indicated that higher NOL10 levels are linked to reduced overall survival times (Fig. 2i), underscoring the potential of NOL10 as a critical prognostic biomarker for prostate cancer.

We also explored how NOL10 expression relates to genome instability in clinical samples. By examining three indicators of genome instability (altered genome fraction, aneuploidy score, and mutation count) in TCGA PRAD samples^34^, we discovered a positive correlation between NOL10 expression and these genomic instability markers (**Supplementary Fig. S1c-e**), further implicating NOL10 in the complexity of prostate cancer pathology.

Given the significant link between the rs4519489 risk allele A and elevated NOL10 expression, alongside the association of NOL10 upregulation with prostate cancer severity, we investigated if the rs4519489 genotype directly impacts prostate cancer patient survival outcomes. Our analysis indicated that the patients carrying the risk genotype A/A at rs4519489 had shorter overall survival times and a higher risk of disease progression (Fig. 2k–m). Furthermore, those carrying the risk genotype with prostate tumors exhibiting higher NOL10 levels showed markedly lower overall and progression-free survival rates compared to patients with A/T or T/T genotypes and lower NOL10 expression (Fig. 2n,o).

In summary, our comprehensive analysis of clinical data demonstrates that the rs4519489 risk allele A and NOL10 expression, either independently or in combination, are associated with aggressive characteristics in prostate cancer. This suggests their viability as biomarkers for assessing disease severity and predicting progression.

### NOL10 as an oncogene potentiates proliferation and metastasis of prostate cancer

**Figure 3:**
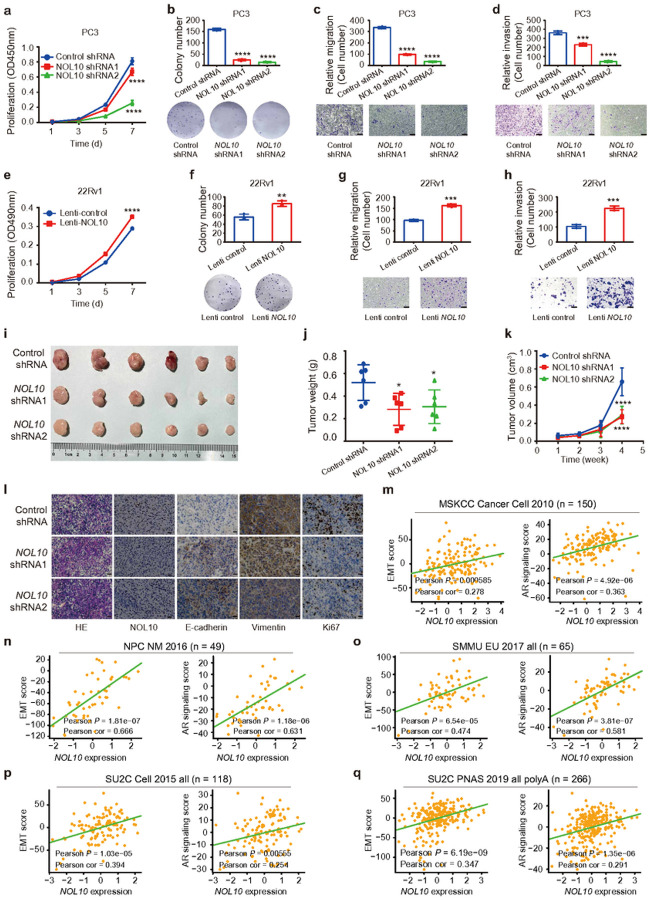
Impact of NOL10 modulation on prostate cancer phenotypes in vivo and in vitro. **a-d**Knockdown of NOL10 in PC3 cells leading to reduced cell proliferation, colony formation, migration, and invasion. **e-h**Overexpression of NOL10 in 22Rv1 cells enhancing cell proliferation, colony formation, migration, and invasion. **i-k**Xenograft experiments showing decreased tumor weight and volume in PC3 cells treated with NOL10-specific shRNAs compared to control. **l**Histological and immunohistochemical analysis of NOL10, E-cadherin, Vimentin, and Ki67 in tumor tissues from nude mice. **m-q**Positive correlation between NOL10 expression and EMT score, as well as AR signaling score, in human prostate cancer tumors across multiple cohorts. *P<0.05, **P<0.01, ***P<0.001, ****P<0.0001; assessed using two-way ANOVA.

We next sought to investigate tumor biology effect of NOL10 in prostate cancer and thus performed shRNA-mediated knockdown of NOL10 in PC3 cells (**Supplementary Fig. S2a, b**). The results showed that the capability of proliferation, colony formation, migration, and invasion of PC3 cells transfected with NOL10 shRNAs were significantly reduced compared with control shRNA transfected cells (Fig. 3a–d). Furthermore, we conducted sgRNA mediated knockout assay of NOL10 in PC3 cells and confirmed the successful knockout efficiency through RT-qPCR and Western blot (**Supplementary Fig. S2c, d**). Subsequent cellular biology assays (CCK8, colony formation, trans-well with or without Matrigel) yielded results aligning with the NOL10 shRNA knockdown findings (**Supplementary Fig. S2e-h**), further underscoring significant influence of NOL10 on tumor cell behavior.

To further substantiate the oncogenic properties of NOL10 in prostate cancer, we employed a doxycycline (Dox)-inducible lentiviral system for overexpressing NOL10 in 22Rv1 cells (**Supplementary Fig. S2i, j**). The cellular function assays revealed that NOL10 overexpression significantly increased oncogenic behaviors, including proliferation, colony formation, migration, and invasion, compared to controls (Fig. 3e–h). Complementing this, shRNA-mediated knockdown of NOL10 in 22Rv1 cells replicated the inhibitory effects on oncogenic activities previously seen in PC3 cells, reinforcing NOL10’s critical contribution to oncogenic traits in prostate cancer (**Supplementary Fig. S2k-p**).

Building on the significant link between NOL10 expression and prostate cancer proliferation, we expanded our study to assess the impact of NOL10 on tumor growth in vivo. We injected nude mice with PC3 cells in which NOL10 expression was diminished through shRNA-mediated knockdown. The results indicated that tumors from the NOL10 knockdown group were markedly smaller in both volume and weight compared to those from the control group (Fig. 3i–k). Histological examination with Hematoxylin and Eosin (H&E) staining demonstrated that the NOL10 knockdown tumors had cells with notably smaller nuclei and fewer atypical features than control tumors (Fig. 3l). Immunohistochemical (IHC) analysis reinforced these findings, revealing increased E-cadherin expression and decreased Vimentin expression in the NOL10 knockdown tumors, indicating a shift towards epithelial characteristics and reduced mesenchymal traits (Fig. 3l). Furthermore, Ki67expression, which signals cell proliferation, was notably lower in the NOL10 knockdown tumors (Fig. 3l), supporting the role of NOL10 in promoting tumor growth and suggesting its potential as a target for therapeutic intervention.

Recognizing the critical importance of epithelial mesenchymal transition (EMT) and androgen receptor (AR) signaling in the progression of prostate cancer, we explored the relationship between NOL10 expression and EMT or AR signaling activity in patients. Through a detailed analysis spanning multiple cohorts, including MSKCC^40^, NPC^42^, SMMU^43^, and SU2C^44^, we consistently found a positive correlation between elevated NOL10 expression and higher EMT or AR signaling scores (Fig. 3m–q and **Supplementary Fig. S2q-s**), highlighting a potential role of NOL10 in modulating key pathways involved in prostate cancer advancement.

Collectively, our results underscore the significant contribution of NOL10 to promoting key oncogenic activities in prostate cancer, both in vitro and in vivo. NOL10 notably boosts cell proliferation, migration, and invasion, and markedly amplifies the EMT process in subcutaneous tumor models in nude mice, underscoring its importance in cancer progression.

### NOL10 promotes cell cycle progression contributing to prostate cancer severity

**Figure 4:**
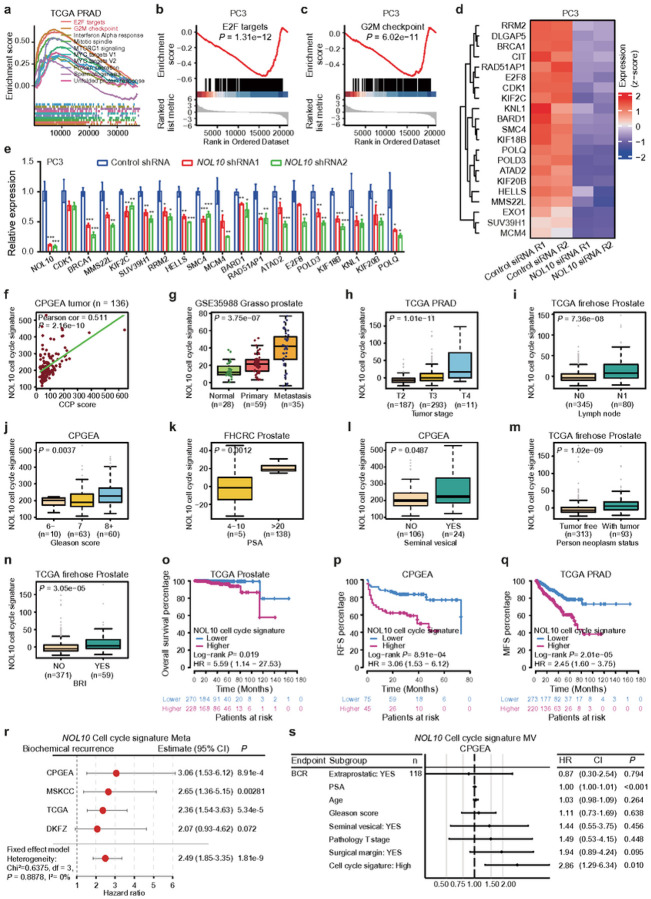
Association of NOL10 gene signature with prostate cancer progression in clinical settings. **a** GSEA analysis in the TCGA prostate cohort, ranking NOL10 expression against HALLMARK collection pathways. **b,c** GSEA on RNA-seq data from NOL10-knockdown PC3 cells, highlighting increased E2F and G2M gene set expression. **d**Heatmap depicting changes in cell cycle-related gene expression from RNA-seq data following NOL10 siRNA knockdown. **e**RT-qPCR validation confirming results from (**d**) using NOL10 shRNA knockdown assay. **f-n** Correlation between NOL10 cell cycle signature (CCS) score and various clinical parameters: cell cycle progression (CCP) score, tumor metastasis, T stage, lymph-node metastasis, Gleason score, PSA level, seminal vesical, person neoplasm status, or biochemical recurrence indicator (BRI) in different prostate cancer cohorts. **o-q**Kaplan-Meier curves showing relationships between overall survival (OS), recurrence-free survival (RFS), and metastasis-free survival (MFS) with the NOL10 cell cycle signature in prostate cancer patients; analyzed using the log-rank test. **r** Forest plots for meta-analysis of hazard ratio estimates of NOL10 CCS for biochemical recurrence-free survival across multiple prostate cancer cohorts. Horizontal error bars represent 95% CIs with HR as the center measure. P values calculated using a two-way Fixed-Effects Model. **s**Multivariate analysis (MV) of BCR in prostate cancer patients, including NOL10 cell cycle signature as a factor in the CPGEA cohort. *P<0.05, **P<0.01, ***P<0.001, ****P<0.0001; assessed using two-way ANOVA.

We next sought to elucidate the potential mechanisms through which NOL10 contributes to prostate cancer progression, and began with a gene set enrichment analysis (GSEA) using the TCGA PRAD dataset, which showed NOL10 expression significantly associated with critical cell cycle pathways, notably E2F targets and G2M checkpoint pathways (Fig. 4a). Subsequently, to assess the impact of NOL10 knockdown on downstream gene expression, we performed RNA sequencing analysis to identify differentially expressed genes (DEGs) in PC3 cells treated with control siRNA or siRNA targeting NOL10 (**Supplementary Fig. S3a**). Our analysis revealed a substantial correlation between two technical replicates, identifying 267 genes as upregulated and 402 genes as downregulated upon NOL10 knockdown (**Supplementary Fig. S3b-d and Supplementary Dataset 2**). Furthermore, GSEA of these downregulated DEGs highlighted their significant enrichment in cell cycle pathways for the NOL10 knockdown group (Fig. 4b, c). Confirmatory RT-qPCR analysis on selected DEGs from the cell cycle pathway validated these RNA sequencing findings (Fig. 4d, e), reinforcing the role of NOL10 in modulating cell cycle-related gene expression in prostate cancer.

To assess the clinical impact of NOL10 target genes in prostate cancer, we developed a cell cycle signature (CCS) based on these genes. Our analysis showed that the NOL10 CCS positively correlates with cell cycle progression (CCP) scores across diverse cohorts, including CPGEA^33^, TCGA PRAD^34^, SU2C^44^, FHCRC^45^, and GSE62872^35^ (Fig. 4f and **Supplementary Fig. S4a-d**). Further, we discovered that the NOL10 CCS was significantly higher in metastatic prostate cancer compared to normal prostate glands and primary tumors (Fig. 4g). Additionally, an elevated NOL10 CCS was linked to more aggressive prostate cancer features, such as advanced T stage, lymph node metastasis, higher Gleason scores, increased PSA levels, seminal vesical invasion, person neoplasm status, and biochemical recurrence indicator (BRI) in various cohorts (Fig. 4h–n and **Supplementary S4e-m**). Importantly, a higher NOL10 CCS also predicted with poorer patient survival outcomes, including overall, recurrence-free, and metastasis-free survival (Fig. 4o–q and **Supplementary S4n-p**), underscoring the potential of the NOL10 CCS as a prognostic marker for prostate cancer aggressiveness and patient prognosis.

To validate the strength of the observed associations, we performed a comprehensive meta-analysis assessing the correlation between the NOL10 CCS and survival outcomes in prostate cancer patients across various cohorts. Our findings demonstrated that a higher NOL10 CCS significantly correlates with shorter biochemical recurrence-free and overall survival (OS) (Fig. 4r and **Supplementary S4q**). Furthermore, intriguingly, multivariate analyses revealed that an elevated NOL10 CCS serves as an independent risk factor for both biochemical recurrence-free survival and OS across multiple cohorts (Fig. 4s and **Supplementary S5a-c**), reinforcing the prognostic value of the NOL10 CCS in predicting outcomes for prostate cancer patients.

In summary, our research indicates that NOL10 potentially regulates genes crucial to cell cycle pathways, with a significant correlation observed between NOL10 target genes and prostate cancer progression, highlighting its importance in promoting the advancement of prostate cancer.

### Unbiased proteomics approach identified USF1 as an allele-specific mediator between rs4519489 and NOL10

**Figure 5:**
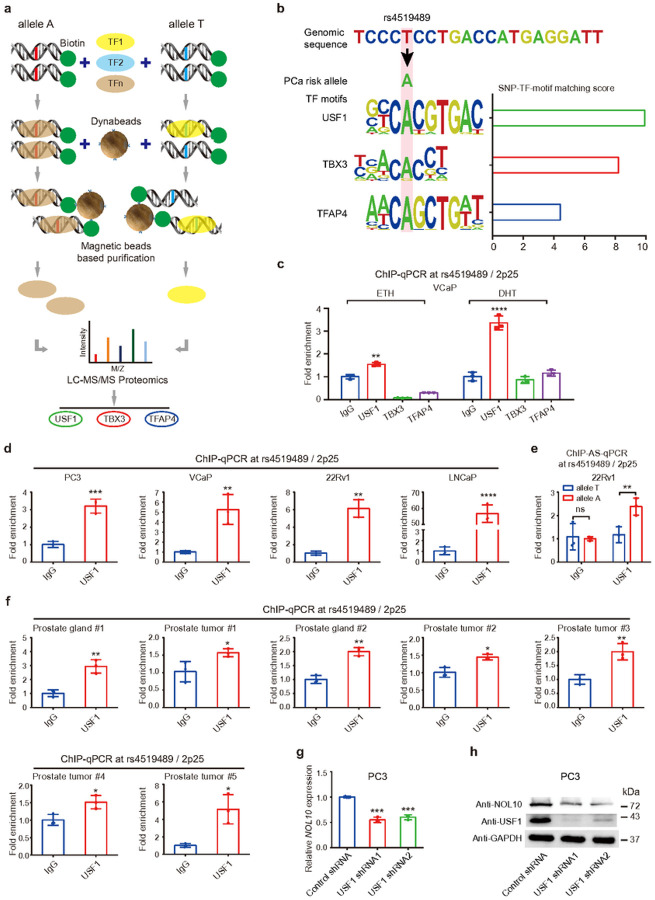
Unbiased proteomics identification of USF1 as interacting transcription factor with the rs4519489 enhancer region. **a**Outline of the proteomics screening method to identify transcription factors interacting rs4519489 region. **b**Enhanced Element Locater (EEL) analysis showing matching scores between the rs4519489 A or T allele and motifs of potential interacting transcription factors. **c**ChIP-qPCR validation confirming the binding of transcription factor (USF1, TBX3, or TFAP4) at the rs4519489 region in VCaP cells treated with ETH or DHT. **d**ChIP-qPCR analysis confirming USF1 binding at the rs4519489 region across PC3, VCaP, 22Rv1, and LNCaP cell lines. **e** ChIP allele-specific qPCR (ChIP-AS-qPCR) demonstrating allele-specific binding of USF1 at rs4519489 in 22Rv1 cells. **f**ChIP-qPCR results indicating USF1 binding at rs4519489 in both normal prostate glands and tumor tissues. **g-h**RT-qPCR and western blot analyses showing decreased NOL10 expression following USF1 knockdown in PC3 cells.

Given the established role of regulatory SNPs in modulating disease susceptibility via alterations in transcription factor (TF)-DNA binding^46^, we further sought to identify TFs that might account for binding differences between the T and A alleles of rs4519489. We thus employed a proteome mass spectrometry approach inspired by the proteome-wide analysis of SNPs (PWAS) technique^47^ (Fig. 5a). By comparing mass spectrometry data for both alleles of rs4519489, we discovered that several TFs, notably USF1, TBX3, and TFAP4, showed specific interactions with the A allele, suggesting their potential roles in mediating the allele-specific effects on gene expression and prostate cancer progression.

We further explored if rs4519489 directly influences the DNA binding affinity of any specific TFs identified in our proteomics study. Utilizing computational analysis with the enhancer element locator (EEL) algorithm^48^ and integrating it with DNA binding position weight matrix data for human TFs^49^, we found that rs4519489 resides within the binding motifs of USF1, TBX3, and TFAP4. Notably, USF1 was identified as the most significant among them (Fig. 5b). This suggests a pivotal interaction between rs4519489 and key TFs, especially USF1, potentially clarifying how this SNP contributes to the genetic risk of prostate cancer.

To validate the binding of TFs to the rs4519489 locus, we performed ChIP-qPCR assays using the antibodies against USF1, TBX3, or TFAP4. Remarkably, the results revealed a significant enrichment of USF1 at the 2p25/rs4519489 locus compared to the IgG control, under both ETH and DHT treatments (Fig. 5c), consistent with our EEL analysis predictions. Moreover, extending this analysis to various prostate cancer cell lines, including PC3, VCaP, 22Rv1, and LNCaP, yielded similar ChIP-qPCR results, confirming significant USF1 enrichment at the rs4519489 locus (Fig. 5d). These results collectively support the hypothesis that USF1, among other TFs, plays a pivotal role in binding to the rs4519489 locus, indicating its involvement in the regulatory processes governing gene expression linked to prostate cancer pathogenesis at this genomic site.

To elucidate the allele-specific binding differences of rs4519489 with USF1, we assessed the genotypes of rs4519489 in five prostate cancer cell lines. Sanger sequencing unveiled that only the 22Rv1 cell line was heterozygous, harboring both A and T alleles (**Supplementary Fig. S6**). Subsequently, ChIP-AS-qPCR targeting rs4519489 demonstrated a notably higher enrichment of USF1 at the A allele compared to the T allele (Fig. 5e). To corroborate these findings in vivo, we conducted ChIP-qPCR assays in normal prostate or tumor tissues using USF1 antibody or IgG control. The qPCR results affirmed the enrichment of USF1 at the rs4519489 region in prostate specimens (Fig. 5f), indicating a potential role of USF1 in regulating NOL10 expression in clinical settings.

Further investigating the regulatory effect of USF1 on NOL10, we initially established a stable PC3 cell line with USF1 shRNA knockdown. RT-qPCR and Western blot analyses revealed a downregulation of NOL10 expression following USF1 knockdown (Fig. 5g–h and **Supplementary S7a**). Additionally, we generated a USF1 sgRNA knockout stable cell line in 22Rv1 cells. Western blot results validated a significant decrease in NOL10 expression following USF1 knockout (**Supplementary Fig. S7b**), consistent with the knockdown assay results in PC3 cells. Further validation was conducted by transiently transfecting 22Rv1 cells with a USF1 overexpression plasmid or an empty vector. Western blot analysis demonstrated elevated NOL10 expression levels in the USF1 overexpression samples compared to the empty vector controls (**Supplementary Fig. S7c**). These experiments were replicated in LNCaP cells, yielding consistent results (**Supplementary Fig. S7d**).

Taken together, our unbiased allele-specific proteomics analysis identified USF1 as a TF interacting with the rs4519489 regulatory region, independent of androgen signaling pathways. USF1 exhibited a preference for the A allele of rs4519489 and positively regulated the expression of NOL10 at both the mRNA and protein levels.

### USF1 positively correlates with NOL10 expression and functions as an oncogene in prostate cancer

**Figure 6:**
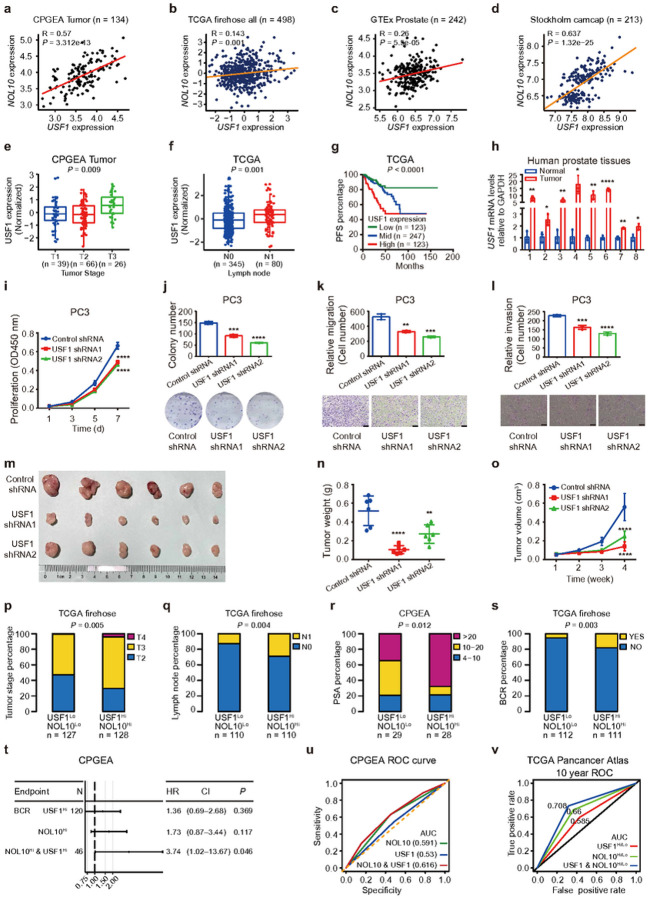
Correlation of USF1 with NOL10 expression and their combined effect on prostate cancer progression. **a-d**Correlation of mRNA expression between NOL10 and USF1 in prostate cancer patients across various cohorts. **e-g**Association of higher USF1 expression with lymph node-positive status, advanced tumor stage, and reduced progression-free survival in prostate cancer. **h**RT-qPCR analysis of USF1 expression in eight prostate cancer versus paracancerous tissue pairs, with GAPDH as a loading control. **i-l**Inhibition of prostate cancer cell proliferation, colony formation, migration, and invasion in PC3 cells following USF1 knockdown. **m-o**Changes in tumor weight and volume in xenograft models using PC3 cells treated with control or USF1-specific shRNAs. **p-s**Comparison of tumor stage, lymph node metastasis, PSA levels, and biochemical recurrence (BCR) between groups with low and high co-expression of NOL10 and USF1 in CPGEA and TCGA cohorts. **t**Combined impact of NOL10 and USF1 on BCR in CPGEA patients. HR: hazard ratio, CI: confidence interval, P: p value. **u-v**Receiver Operating Characteristic (ROC) curves predicting survival in prostate cancer patients, illustrating the combined effect of NOL10 and USF1 in CPGEA and TCGA cohorts.

To explore the association between USF1 and NOL10 expression, we conducted a comprehensive analysis across multiple datasets, revealing a consistent positive correlation between the mRNA expression levels of USF1 and NOL10. This correlation was observed in diverse cohorts, including CPGEA^33^, TCGA PRAD^34^, GTEx^50^, Stockholm camcap^51^, SMMU^43^, and NPC cohorts^42^ (Fig. 6a–d and **Supplementary S7e, f**), indicating a potential role for USF1 in upregulating NOL10 expression in clinical contexts.

Further investigating the clinical relevance of USF1 expression, we analyzed two large-scale clinical datasets. In the CPGEA cohort^33^, high USF1 expression showed a significant association with advanced tumor stages in prostate cancer (Fig. 6e). Similarly, analysis of the TCGA PRAD cohort^34^ revealed that elevated USF1 expression was significantly correlated with malignant characteristics of prostate cancer, including tumor stage, lymph node metastasis, Gleason score, biochemical recurrence, person neoplasm status, and progression-free survival (Fig. 6f–g and **Supplementary S7g-j**). These findings underscore the potential prognostic value of USF1 expression in prostate cancer.

To validate our findings from the clinical databases, we conducted RT-qPCR verification using eight pairs of prostate cancer tissues and their adjacent normal tissues from the CPGEA cohort. This verification reinforced our database analysis, showing higher expression of USF1 in prostate cancer tissues compared to adjacent normal tissues (Fig. 6h). These results collectively underscore the significant correlation between USF1 expression and prostate cancer progression, highlighting USF1 as a potential biomarker for disease severity and as a target for therapeutic intervention.

Recognizing the pivotal role of TFs in cancer development^52^, and considering the regulatory influence of USF1 on the oncogene NOL10, we hypothesized that USF1 might possess critical biological functions in prostate cancer. To test this hypothesis, we established a stable PC3 cell line with shRNA-mediated USF1 knockdown (**Supplementary Fig. S8a, b**). Subsequently, we performed a series of tumor cell biology assays, including CCK8 cell proliferation, colony formation, and cell migration and invasion experiments. The results indicated that, compared to the control shRNA group, the USF1 knockdown group exhibited significantly reduced cell proliferation, colony formation, migration, and invasion abilities (Fig. 6i–l). In addition, we generated a stable 22Rv1 cell line overexpressing USF1 and conducted similar cell function experiments (**Supplementary Fig. S8c, d**). The outcomes of these experiments revealed that cell proliferation, colony formation, migration, and invasion in the USF1 overexpressed 22Rv1 cells were significantly enhanced compared to the cells with the empty vector control (**Supplementary Fig. S8e-h**). These findings provide compelling evidence that USF1 plays a critical role in the modulation of prostate cancer cell behaviors, potentially driving the progression and aggressiveness of the disease.

To validate our in vitro findings in an in vivo setting, we carried out subcutaneous tumor transplantation experiments using nude mice. These mice were injected subcutaneously with PC3 cells control stably transduced with either control or USF1 target shRNAs. The results showed that both the volume and weight of the tumors in the USF1 knockdown groups were significantly reduced compared to the control group (Fig. 6m–o). Additionally, histopathological examination, including H&E staining and IHC analysis of the tumor tissues from the USF1 knockdown groups, displayed similar patterns to those observed in the NOL10 knockdown group, showing a diminished capacity for subcutaneous tumor formation and inhibition of the epithelial-mesenchymal transition (EMT) process in the tumors (Fig. 3l and **Supplementary Fig. S8i**).

In summary, our comprehensive analysis demonstrates a positive correlation between USF1 and NOL10 expression, with clinical data indicating a connection between USF1 and malignant characteristics of prostate cancer. Furthermore, our findings demonstrate that USF1 enhances the aggressiveness of prostate cancer cells in vitro and promotes tumor formation and the EMT process in vivo in mice.

### Combined effects of NOL10 and USF1 on prostate cancer progression

To explore the combined impact of NOL10 and USF1 on prostate cancer progression, we conducted an analysis of their synergistic expression and its correlation with clinical pathology characteristics. Using data from both the CPGEA and TCGA datasets, we found that patients with elevated co-expression levels of NOL10 and USF1 showed a significant association with increased tumor stage, lymph node metastasis, PSA levels, Gleason score, and biochemical recurrence (Fig. 6p–s and **Supplementary Fig. S9a,b**). This suggests that the joint expression of NOL10 and USF1 could serve as a potential biomarker for assessing disease severity and progression in prostate cancer. The observed correlation underscores the importance of these two molecular entities in the pathophysiology of the disease and highlights their potential as targets for therapeutic intervention.

To further understand the clinical implications of NOL10 and USF1 co-expression in prostate cancer, we calculated hazard ratios (HR) for biochemical recurrence, metastasis, and overall survival based on the levels of NOL10 and USF1 expression across several cohorts, including CPGEA^33^, TCGA^34^, and SU2C^44^. The results consistently showed that higher co-expression of NOL10 and USF1 was associated with increased hazard ratios in these cohorts (Fig. 6t and **Supplementary Fig. S9c-e**), indicating that patients with elevated levels of both NOL10 and USF1 expression are at a greater risk of disease progression.

To further evaluate the predictive power of NOL10 and USF1 expression in prostate cancer prognosis, we constructed time-independent Receiver Operating Characteristic (ROC) curves. These analyses demonstrated that the combined effect of NOL10 and USF1 outperformed the predictive accuracy of either gene alone. Moreover, time-dependent ROC curves were generated to assess the predictive capability for 1-, 3-, 5-, and 10-year survival outcomes. These analyses indicated that the combination of NOL10 and USF1 offered superior prognostic prediction over either gene alone across various cohorts, including CPGEA^33^, TCGA^34^, SU2C^44^, and DKFZ^53^ (Fig. 6u–v and **Supplementary S10a-l**).

**Figure 7:**
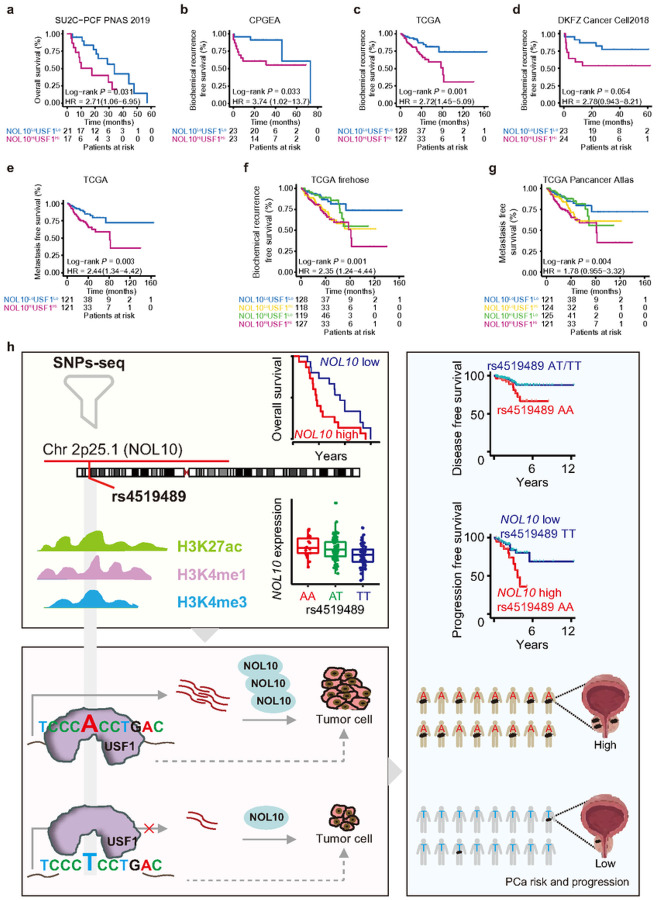
Joint influence of NOL10 and USF1 on prostate cancer progression in clinical settings. **a-g**Correlation of combined NOL10 and USF1 expression with overall survival (OS), biochemical-recurrence-free survival (BFS), or metastasis-free survival (MFS) in prostate cancer patients across various cohorts. Hi, higher; Lo, lower. **h**Schematic model illustrating how the 2p25 locus interaction with USF1 regulates NOL10, thereby driving prostate cancer cell growth and increasing tumor severity.

Furthermore, we explored the combination effect of NOL10 and USF1 expression on the prognosis of prostate cancer patients. Our analysis revealed that the synergistic co-overexpression of NOL10 and USF1 was associated with poorer overall survival, biochemical recurrence-free survival, and metastasis-free survival in patients with prostate cancer, consistently across multiple cohorts including CPGEA^33^, TCGA^34^, SU2C^44^, and DKFZ^53^ (Fig. 7a–g). These findings underscore the significant prognostic value of assessing both NOL10 and USF1 expression levels in prostate cancer patients. The synergistic effect of their co-overexpression serves as a robust indicator of disease progression and patient outcomes, highlighting their potential as critical biomarkers in the clinical management and treatment of prostate cancer.

## Discussion

In this study, we have revealed the regulatory relationships among the prostate cancer risk locus rs4519489, USF1, and NOL10 (Fig. 7h). By integrating high-throughput SNPs-seq and unbiased proteomics, we uncovered the prostate cancer risk SNP rs4519489 (2p25) within a functional enhancer, where USF1 exhibits a preference for binding the risk allele A, thereby upregulating NOL10. This highlights a direct regulatory pathway mediated by USF1 at this specific genomic locus. Moreover, NOL10 is implicated in the regulation of cell cycle pathways, thereby facilitating the progression of prostate cancer supported by cell line and mouse model experiments. Notably, both NOL10 and USF1 are linked to aggressive prostate cancer phenotypes, underscoring their clinical relevance as potential prognostic markers and therapeutic targets.

Identifying functional causal SNPs and understanding their biological roles within hundreds of GWAS-reported risk loci remains a formidable challenge^12, 20, 54^. While various methods have been developed to address this, there is an ongoing need for more comprehensive studies to bridge the gap between GWAS findings and disease mechanisms^17, 55–57^. In the context of prostate cancer, our team introduced an advanced approach called SNPs-seq^29^, designed for high-throughput screening of SNPs for allele-specific protein binding differences. One notable finding was rs4519489 in the 2p25 locus, which exhibited significant protein binding bias between its A and T alleles. Moreover, rs4519489 showed strong LD with two GWAS lead SNPs, rs9287719^31, 32^ and rs1990613^14, 15^ suggesting a robust association with prostate cancer susceptibility and indicating its potential role in disease etiology and progression.

Herein we conducted a comprehensive investigation to validate the allele-specific protein binding and regulatory function of rs4519489, along with its clinical implications. Our eQTL analysis, using data from the CPGEA cohort, revealed a significant association between the rs4519489 A/A risk genotype and increased expression of NOL10. While the roles of NOL10 in cancer have been underexplored, previous studies identified it as an essential nucleolar protein crucial for maintaining nucleolar structural integrity^58, 59^. To elucidate its potential oncogenic role, we performed functional analyses demonstrating NOL10 status as a novel oncogene with prognostic potential in prostate cancer. Mechanistically, NOL10 likely influences the expression of genes associated with critical cell cycle pathways, including E2F targets and the G2M checkpoint. These findings collectively suggest that NOL10 actively contributes to prostate cancer progression rather than being a passive bystander. Its ability to modulate key cellular processes central to cancer development underscores its potential as a therapeutic target in prostate cancer intervention.

Our study refined an allele-specific proteomics screening method to investigate how SNPs can influence gene expression by modulating the binding affinity of key TFs. Analytical outcomes indicated that USF1 is the most likely TF to mediating the genetic effect of the rs4519489/2p25 locus. We confirmed USF1 chromatin occupancy at the rs4519489 site and its positive regulation of NOL10 expression, linking USF1 for the first time with the genetic predisposition to prostate cancer. Moreover, USF1 was significantly associated with malignant characteristics of prostate cancer, as evidenced by clinical data showing correlations with higher tumor stages, lymph node metastasis, elevated Gleason scores, biochemical recurrence, and poorer progression-free survival. Additionally, analysis of clinical prostate cancer samples revealed higher USF1 expression in tumor tissues compared to normal prostate tissues. These findings collectively suggest that USF1 not only serves as a potential biomarker for prostate cancer severity but also actively promotes disease progression. Its ability to drive tumorigenesis and influence key cancer cell behaviors underscores its potential as a therapeutic target in prostate cancer intervention.

In summary, our study unveils a pivotal regulatory mechanism underlying prostate cancer pathogenesis, centered around the genetic risk variant rs4519489 at the 2p25 locus. We demonstrate that this region acts as an enhancer, modulating the binding affinity of the newly identified regulator USF1. This regulatory shift subsequently governs the expression of NOL10, a key contributor to prostate cancer progression. By delving into the functional aspects of the 2p25/NOL10 genetic risk locus, we significantly enhance our understanding of prostate cancer development. Our findings highlight the importance of rs4519489 and NOL10 in the molecular landscape of prostate cancer, offering potential as both a diagnostic biomarker and a therapeutic target. Targeting the regulatory axis involving rs4519489, USF1, and NOL10 holds promise for innovative therapeutic strategies aimed at curtailing prostate cancer progression and severity.

## Methods

### Ethics Statement

The utilization of clinical human specimens in our study, along with the meticulous review of relevant patient records, received the endorsement of the Ethical Committee and Institutional Review Board of the School of Basic Medical Sciences at Fudan University (Approval number: 2021–005). All procedures involving human samples were conducted in strict adherence to the ethical guidelines set forth in the Declaration of Helsinki. Informed consent was duly obtained from each participating patient, ensuring the utmost respect for patient confidentiality throughout the study.

Furthermore, all animal experiments conducted as part of this study were approved by the Animal Care and Use Committee of the School of Basic Medical Sciences at Fudan University (Ethical approval number: 20200713–002). These experimental protocols were rigorously aligned with the Guide for the Care and Use of Laboratory Animals, underscoring our commitment to the ethical and humane treatment of all animals involved in our research endeavors. This compliance is a testament to our dedication to maintaining the highest standards of ethical conduct in all aspects of our research.

### Tissue samples

Tissue samples employed in our study were meticulously selected to provide robust insights into the molecular mechanisms underlying prostate cancer. For ChIP-seq of histone modifications, including H3K27ac, H3K4me1, and H3K4me3, we utilized both normal and tumor prostate tissues from the CPGEA cohort^33^. For USF1 ChIP assays, we collected chromatin from normal prostate as well as prostate tumor tissues obtained from the FUSCC (Fudan University Shanghai Cancer Center) cohort^38^. Furthermore, to validate the expression levels of NOL10 and USF1 in patient tissues, we extracted RNA from five tissue pairs comprising prostate tumor tissues and their adjacent normal counterparts. We also isolated protein samples from two of these tissue pairs, all of which were acquired from the FUSCC cohort.

### Mice

Male nude mice aged 6 weeks were acquired from Gempharmatech Company, China, for conducting in vivo experiments. The mice were maintained under controlled environmental conditions to ensure their wellbeing and the validity of our experimental outcomes. The housing conditions included a 12-hour light-dark cycle, with the mice accommodated in sterilized plastic cages. The ambient temperature of housing facility was regulated between 21.7–22.8 °C, and the humidity was maintained within a range of 40–60%. To ensure the highest standards of hygiene and health, the water provided to the mice was autoclaved, and their cages were replaced once every week. The health and wellbeing of the mice were continuously monitored through a dirty bedding sentinel program, which is a well-established method for detecting health issues in laboratory animals. For all in vivo studies conducted as part of this research, we adhered to a protocol that included cohorts of three or more mice per experimental group. This approach was designed to ensure the reliability and reproducibility of our results. The experiments were repeated two to three times independently, further strengthening the robustness of our findings.

### Cell lines

The human prostate cancer cell lines, including PC3 (#CRL-1435), DU145 (#TCHu222), 22Rv1 (#TCHu 100), LNCaP (#CRL-1740), VCaP (#TCHu220), and the human embryonic kidney (HEK) 293T (#CRL-11268) were obtained from the American Type Culture Collection (ATCC, USA) and the Cell Bank of the Chinese Academy of Sciences (China). The culture conditions for these cell lines were carefully maintained to ensure their optimal growth and viability. The PC3, 22Rv1, and LNCaP cells were cultured in RPMI 1640 medium, whereas the DU145, VCaP, and HEK 293T cells were grown in DMEM medium. The cell culture media for all these lines supplemented with 10% fetal bovine serum (FBS) (#FSP500, Genetimes Technology) and 1% penicillin/streptomycin (#MA0110, MeilunBio). The cell cultures were housed in a 37 °C incubator with a humidified atmosphere containing 5% CO_2_. To ensure the integrity and reliability of our research, all cell lines underwent regular testing for mycoplasma contamination, with consistently negative results. Additionally, these cell lines have been authenticated by short tandem repeat (STR) fingerprinting.

### Molecular cloning

For construction of shRNA plasmid, primers were designed based on the mRNA sequences of NOL10 (NM_024894.4) and USF1 (NM_007122.5) obtained from the National Center for Biotechnology Information (NCBI). Post primer annealing, the shRNA sequences were cloned into the pLKO.1 puro vector (#8453, Addgene).

For construction of sgRNA plasmid, sgRNA oligos were designed using an online tool (http://crispor.tefor.net). For annealing, we mixed 5 μl sense sgRNA oligos (100 μM) and 5 μl anti-sense sgRNA oligos (100 μM) with 10 μl annealing buffer (5×), and 30 μl ddH_2_O. The oligos were annealed in a thermocycler at 95 °C for 5 minutes, followed by a gradual temperature decrease to 25 °C at a rate of 1 °C/minute. The annealed oligos were then inserted into the Lenti CRISPR V2 Puro vector (#52961, Addgene).

For construction of overexpression plasmid, the coding regions of NOL10 or USF1 were amplified from mixed cDNA obtained from prostate cancer cells. The amplified products were cloned full-length into the pcDNA3.1 V5 vector (#V81020, Thermo Fisher Scientific) or Lenti-X Tet-One Inducible Puro V5 vector (modified from vector of #631847, Takara Bio). This was achieved using either restriction enzymes or homologous recombination techniques.

Details of the primer sequences utilized were provided in **Supplementary Table 1**.

### Electrophoresis mobility shift assay (EMSA)

We employed an electrophoresis mobility shift assay (EMSA) to validate the allele-dependent protein binding differences. This assay was performed using the LightShift Chemiluminescent EMSA Kit (#20148, Thermo Fisher Scientific). The oligonucleotides required for this experiment were synthesized by Tsingke Biotech. The target oligonucleotide, 29 base pairs in length with the SNP positioned centrally, was labeled using the Biotin 3’ End DNA Labeling Kit (#89818, Thermo Fisher Scientific). The nuclear proteins were extracted from LNCaP cells to be used in the binding reactions. The 20 μl reaction mixture included 1x binding buffer, 1 μg of Poly (dI-dC), 1 μl of nuclear extract, a 2-fold or 200-fold excess of unlabeled oligo for competitive assays, and 20 fmol of 3’ end labeled oligo. The reaction mixtures were subjected to electrophoresis on a 6% polyacrylamide gel using 0.5x TBE buffer. Following electrophoresis, the samples were transferred onto a nylon membrane (#77016, Thermo Fisher Scientific). After cross-linking, protein-DNA complexes were detected using the Chemiluminescent Nucleic Acid Detection Module. Visualization was achieved using the Tanon 5200 Imaging System (Tanon, China). The sequences of the oligonucleotides used in the assay are detailed in **Supplementary Table 2**.

### Luciferase enhancer reporter assay

To investigate the regulatory potential of SNP rs4519489, we employed an allele-dependent luciferase reporter assay. This assay involved cloning allele-specific sequences (either the T or A allele, achieved through site-directed mutagenesis) from the genomic DNA of human prostate cancer cells into a firefly luciferase pGL4.23 minimal promoter vector (#E8411, Promega) or the pGL3 promoter vector (#E1761, Promega) to assess enhancer activity. The constructs were transiently transfected into 22Rv1 or LNCaP cells. For hormonal treatment, cells were exposed to either dihydrotestosterone (DHT) or ethanol (ETH). Transfection was facilitated using Lipofectamine 3000 DNA Transfection Reagent (#L3000015, Thermo Fisher Scientific). To normalize the results, we co-transfected cells with the renilla luciferase pGL4.75 plasmid (#E6931, Promega) as an internal control. The experiments were conducted in 96-well plates, with each well containing 100 μl of medium seeded with 3 × 10^5^ 22Rv1 or LNCaP cells/ml. Post-transfection, the cells were incubated at 37 °C in a 5% CO2 atmosphere for 48 hours. The luciferase activity was measured using the Dual Luciferase Reporter Assay System (#E1960, Promega) on a bioluminometer. Each construct was tested in at least three replicate wells. The results were then statistically analyzed using a two-tailed Student’s T-test. Details of the primer sequences, cloning methods, and enzymes used are available in **Supplementary Table 1**.

### CRISPRi

We generated stable PC3 cell lines expressing CRISPR dCas9 KRAB by transfecting cells with the pLX303-ZIM3-KRAB-dCas9 plasmid (#154472, Addgene). Post-transfection, cells underwent antibiotic selection with 6 μg/mL blasticidin for two weeks. Guide RNAs (gRNAs) were specifically designed to target the active epigenetically marked chromatin region encompassing rs4519489. To ensure comprehensive analysis, we included a negative control (scramble sgRNA) and a positive control (HPRT1 promoter targeting gRNA). These gRNA cassettes were synthesized by Tsingke Biotech and subsequently cloned into the pgRNA humanized vector (#44248, Addgene). The PC3 cells stably expressing KRAB-dCas9 were then infected with the gRNA vectors. Following infection, the cells underwent selection with 2 μg/mL puromycin for five days. The primers used for all gRNAs are detailed in **Supplementary Table 3**.

### CRISPR/Cas9 mediated genome editing assay

The cells were seeded in a 6-well plate, ensuring they were at the appropriate density for transduction. The sgRNA lentivirus specific to NOL10 was prepared in advance. Virus Addition: For each well, 1 ml of the lentivirus-containing medium was combined with an equal volume of the cell culture medium. To enhance the efficiency of viral transduction, 10 μg/ml polybrene was added to this mixture. Incubation Period: The cells were incubated for 48 hours to allow sufficient time for the viral transduction to occur. Medium Change and Selection: Post-transduction, the medium in each well was replaced with fresh medium containing 1 μg/ml puromycin. This step was crucial for selecting cells that had successfully incorporated the sgRNA, as puromycin resistance is conferred only to those cells where the viral transduction (and thus the sgRNA incorporation) was successful.

### siRNA and shRNA knockdown assay

PC3 cells were grown to 70–80% confluency for optimal transfection conditions. Cells were transfected with either control siRNA or siRNAs targeting NOL10 using Lipofectamine RNAi MAX Transfection Reagent (#13778150, Thermo Fisher Scientific). The medium was replaced after 12 hours post-transfection, and cells were collected after 48 hours for further analysis. The specific sequences of siRNAs used are detailed in **Supplementary Table 3**.

Lentiviral constructs with shRNA targeting NOL10 or USF1 were produced in 293T cells using a third-generation packaging system. Cells were seeded in a 6-cm dish at 70%–80% confluency a day before transfection. A mix of four plasmids (pCMV-VSV-G, #14888, Addgene; pRSV-Rev, #12253, Addgene; pMDLg/pRRE, #12251, Addgene and the lentiviral target vector) was prepared in a 1:1:1:3 ratio, totaling 10 μg, and diluted in Opti-MEM with PEI reagent. After 24 hours, the medium was replaced with 2 ml fresh medium, and the virus-containing medium was collected every 24 hours for three days, filtered through a 0.45 μm filter, and stored −80 °C. For virus transduction, the desired cells were seeded in a 6-well plate and incubated with the lentivirus-containing medium supplemented with 8 μg/ml polybrene (#TR-1003-G, Sigma). In case of puromycin selection construct, after 24 hours the medium was replaced with pre-warmed medium, and 48 hours after transduction the medium was changed with fresh medium containing puromycin (2 μg/ml; #MA0318, MeilunBio) in a final concentration of 2 μg/ml for selection. Non-transduced cells served as controls for determining cell survival upon puromycin selection.

### Overexpression assay

Both 22Rv1 and LNCaP cells were grown to 70–80% confluency for optimal transfection efficiency. The transfection mixture consisted of the pcDNA3.1 construct, P3000 reagent, Lipofectamine 3000 reagent (#L3000015, Thermo Fisher Scientific), and Opti-MEM (#11058021, Thermo Fisher). The prepared mixture was added to the cells and incubated for 48 hours to allow for gene expression. Post-incubation, cells were harvested for subsequent analyses. For establishing stable overexpression cells, 22Rv1 cells were infected with Lenti-X Tet-One inducible Puro V5 constructs. Post-infection, cells were selected and maintained under appropriate conditions to ensure stable integration and expression of the target gene.

### RNA isolation, reverse transcription, and quantitative PCR

Total RNA was isolated using the EZ-10 DNAaway RNA Mini-Preps Kit (#B618133, Sangon Biotech). 1 ug total RNA was reverse transcribed using the HiScript III RT SuperMix for qPCR kit (#R323–01, Vazyme) and the resulting cDNA was diluted 20 times. RNA expression was quantified using the ChamQ universal SYBR qPCR master mix (#Q711–02, Vazyme) on the Light Cycler 480 (Roche). GAPDH, a stable housekeeping gene, was used as a reference for normalizing gene expression levels in the samples. Each sample was measured in triplicate to ensure the accuracy and reliability of the data. Relative gene expression was calculated using the ΔΔCT (ΔCT [sample] – ΔCT [control average]) method. The sequences of all oligonucleotides used in these procedures are provided in **Supplementary Table 2**.

### Western blot

The cell pellet was resuspended in lysis buffer, followed by centrifugation. The supernatant containing the extracted proteins, was collected. Protein concentrations were determined using the BCA Protein Assay Kit (#P0012S, Beyotime Biotechnology). Equal amounts of protein lysate (30 μg) were denatured using protein loading buffer (#P0015F, Beyotime Biotechnology). The denatured proteins were separated by SDS-PAGE, and transferred to 0.45 μm PVDF membranes (#IPVP00010, Millipore). The membrane was blocked for 1 hour at room temperature using blocking buffer (5% nonfat milk in TBST) while gently shaking. The blocked membrane was incubated overnight at 4 °C with primary antibodies diluted in blocking buffer, under gentle rotation. Post-incubation, the membrane was washed five times for 5 minutes each with TBST. The membrane was then incubated with HRP-conjugated secondary antibody diluted in blocking buffer for 1 hour at room temperature on a rotor. Afterwards, the membrane was washed five times for 5 minutes each using TBST. Finally, the membrane was developed using Omni-ECL Western Blotting Substrate (#SQ202L, Epizyme) or Omni-ECL Femto Maximum Sensitivity Substrate (#SQ201, Epizyme). The developed blot was imaged using the ChemiDoc Imaging System (Bio-Rad). The specific antibodies used in this study are listed in **Supplementary Table 4**.

### Tumor cell biology experiments

For cell proliferation assay, cells were seeded in 96-well plates (1 × 10^3^ cells per well for PC3, 3 × 10^3^ cells per well for 22Rv1 in 100 μl medium). Cell viability and proliferation were measured using CCK 8 Kit (#MA0218, MeilunBio) or MTT (#SY316, Beyotime Biotechnology) kits. Absorbance readings at 450 nm (CCK-8) or 490 nm (MTT) were taken at specific time points. Data, obtained from at least triplicate wells, were analyzed using two-tailed Student’s T-test or two-way ANOVA.

For colony formation assay, cells (1 × 10^3^ for PC3, 4 × 10^3^ for 22Rv1) were seeded in 6-well or 12-well plates. After two weeks, colonies were fixed in 4% paraformaldehyde and stained with crystal violet (#A600331–0100, Sangon Biotech).

For cell migration assay, cells were trypsinized, resuspended in serum-free medium, and 200 μl were placed into 8 μm transwell inserts (#353097, BD). Lower chambers were filled with 600 μl of normal growth medium and cells were incubated for 36 hours. Post-incubation, cells were fixed with 4% formaldehyde and stained with crystal violet.

For cell invasion assay, the transwell inserts were coated with 100 μl Matrigel (#40183ES10, Yeasen) diluted in serum-free medium. Invasive cells on the bottom surface of the filters were counted in five microscopic fields per membrane. Both the migration and invasion assays were statistically analyzed using two-tailed Student’s T-test or two-way ANOVA, with each assay performed in three replicates.

### In vivo nude mice subcutaneous xenograft model

Male nude mice from Gempharmatech Company, China, were randomly divided into different groups, with six mice in each group. Control shRNA, NOL10 shRNA or USF1 shRNA stable PC3 cells were harvested, trypsinized, and washed with PBS. Each mouse received a subcutaneous injection of 5 × 10^6^ PC3 cells in 50 μl PBS mixed with 50 μl Matrigel (#40183ES10, Yeasen) into the right dorsum. Tumor sizes were measured weekly using a vernier caliper, and volumes calculated using the formula: V = 0.5 × (Length × Width^2^). After four weeks, mice were sacrificed, and subcutaneous tumors were removed for further analysis.

### Immunohistochemistry (IHC)

Subcutaneous tumor tissues from each group of mice were collected and fixed in 4% paraformaldehyde, dehydrated, and embedded in paraffin. Paraffin sections (5 μm thickness) were deparaffinized, rehydrated, and stained with haematoxylin and eosin (H&E). Sections underwent hydrogen peroxide treatment, antigen retrieval, and blocking. Overnight incubation with primary antibodies (NOL10, E-cadherin, Vimentin, Ki67 or USF1) at 4 °C was followed by application of biotinylated secondary antibodies and streptavidin conjugated HRP. Detection was developed using DAB substrate solution. Details of the antibodies used are provided in **Supplementary Table 4**.

### RNA-seq and differential expression genes (DEG) analysis

PC3 cells were transfected with either siRNA targeting NOL10 or a negative control siRNA, incubated for 48 hours under standard cell culture conditions, with two biological replicates. Total RNA was extracted using Trizol reagent (#15596018, Thermo Fisher Scientific). RNA-seq libraries were prepared using the Stranded mRNA-seq Lib Prep Module (RK20349, Abclonal). The quality of libraries was assessed using LabChip Touch, and sequencing was conducted at Annoroad Company with Illumina sequencing platforms.

Raw sequence data were preprocessed using FastQC (v.0.11.9) (www.bioinformatics.babraham.ac.uk/projects/fastqc/) for quality assessment. AdapterRemoval (v.2.3.2)^60^ was used for quality trimming and adapter removal with default parameters. The processed reads were aligned to the human genome (hg38) using STAR (v.2.7.9a)^61^ and the aligned BAM files were sorted using SAMtools (v.1.13)^62^. HTSeq (v.0.13.5)^63^ was employed to quantify aligned sequencing reads against UCSC gene annotation with the parameters “-s reverse, -i gene_id”. DESeq2 (v.1.30.1)^64^ was used for DEG analysis from the read count matrix. Genes with low expressions (<5 cumulative read count across samples) were filtered out. An adjusted P value < 0.05 was applied to generate the list of differentially expressed genes. DEGs were ranked according to their fold change. Statistical tests were applied to control or treatment to ensure high correlations between technical replicates. Data normalization was performed using the variance Stabilizing Transformation (VST) method. A heatmap presenting DEGs between siRNA control and siRNA NOL10 samples was generated using the R package “pheatmap” (v.1.0.12). Detailed information about the software and algorithms used is provided in **Supplementary Table 5**.

### Gene set enrichment analysis (GSEA)

We applied GSEA (v.4.0.3) to interpret the RNA-seq results of NOL10 knockdown. A pre-ranked gene list was compiled by calculating data following the formula sign (logFC) *-log (p value), and the data were sorted in a descending order. The GSEA Preranked test was used to test the enrichment of phenotypic genes in Hallmark gene sets (H collection). Parameters were set as follows: Enrichment statistic = “weighted”, Max size (exclude larger sets) = 5000, number of permutations = 1000. All other parameters remained as default. GSEA enrichment plots were generated using R packages “clusterProfiler” (v.3.14.3)^65^ and “enrichplot” (v.1.12.0). The software and algorithms were listed in **Supplementary Table 5**.

### Allele specific unbiased proteomics screening

To determine the transcription factors (TFs) contributing to the allelic binding difference of rs4519489, we adapted the PWAS (Proteome Wide Analysis of SNPs) mass spectrometry method^46^. This modification enabled us to identify specific TFs that preferentially bind to different alleles of rs4519489. Firstly, we synthesized a 29-base pair oligonucleotide containing either the T or A allele of rs4519489. The oligonucleotide was labeled using the Biotin 3’ End DNA Labeling Kit (#89818, Thermo Fisher Scientific). Secondly, the biotin-ds-oligos were incubated with freshly prepared nuclear extract from LNCaP cells using NE-PER nuclear and cytoplasmic extraction reagents (#78833, Thermo Fisher Scientific). The binding reactions (total 100 μl) of DNA and nuclear protein included 54 μl ultrapure water, 10 μl binding buffer at 10x, 5 μl poly(dI•dC) of 1 μg/μl, 20 μl nuclear extract, 1 μl proteinase inhibitor, and 10 μl biotin-ds-oligos were incubated at room temperature for 15 minutes. Thirdly, the Dynabeads M280 streptavidin (#11205D, Thermo Fisher Scientific) were washed three times with washing buffer, and then incubated with the biotin-ds-oligos-nuclear protein complex for 20 minutes at room temperature. The complex was washed five times using a magnetic stand and resuspended in 50 μl of 50 μM ammonium bicarbonate buffer. Finally, the allele-specific complexes (for alleles T and A of rs4519489) were analyzed using a LC-MS/MS mass spectrometer (LTQ XL, Thermo Fisher Scientific). The sequences of oligonucleotides used in this allele-specific unbiased proteomics screening are detailed in **Supplementary Table 2**.

### Chromatin immunoprecipitation (ChIP)

PC3, 22Rv1, LNCaP, and VCaP cells were cross-linked with 1% formaldehyde for 10 minutes and fixation was stopped with 125 mM Glycine at room temperature for 5 minutes with gentle shaking. Cell pellets were suspended in hypotonic lysis buffer (with protease inhibitor cocktail) for 45 minutes. Nuclei were washed with cold PBS and re-suspended in SDS lysis buffer (final 0.5% SDS). Chromatins was sonicated to ~400 bp for ChIP-qPCR and ChIP-AS-qPCR, and ~200 bp for ChIP-seq (Diagenode bioruptor or Covaris M220). Dynabeads Protein G (#10004D, Thermo Fisher Scientific) were washed twice by blocking buffer, and then incubate the beads with antibodies (6 μg for TF and 2 μg for histone modification antibodies) at 4 °C overnight. The sonicated chromatin (300 μg for TF, and 20 μg for histone ChIP assay) was diluted in IP buffer to final volume of 1.3 ml, then added to 40 μl of Dynabeads antibody complex. After incubation overnight at 4 °C, the complex was washed six times with washing buffers. The DNA protein complex will be separated from beads by extraction buffer. DNA-protein complexes were reverse cross-linked with Proteinase K and NaCl at 65 °C overnight. The DNA was purified using the MinElute PCR Purification Kit (#28006, Qiagen).

For tissue ChIP assay, the samples were cut into small pieces by tiny scissors, fixed in 1.5% formaldehyde for 10 minutes at room temperature, and then quenched with Glycine. The tissues were mechanically extracted by applying 8 cycles using a tissue freezing grinder (Jingxin, China). To isolate nuclei, we suspended the tissue pellet in hypotonic lysis buffer (with DTT and protease inhibitor cocktail) for 40 minutes at 4 °C. The tissue mass was filtered out with a sterile 100 μm filter. Chromatin was sheared to 200–500 bp using a high power Bioruptor plus sonicator or Covaris. For each ChIP, the chromatin (30 μg for a TF and 1.5 μg for a histone modification ChIP assay) were incubated with antibodies (4 μg for TF and 2 μg for histone) overnight at 4 °C. The antibody chromatin complex were conjugated with washed Protein G Dynabeads overnight at 4 °C. The 100 ul eluted chromatin protein complex were reverse cross-linked by adding 6 μl of 5M NaCl and 5 ul of Proteinase K and then incubating overnight at 65 °C. The immunoprecipitated and input DNA was purified using the MinElute PCR Purification Kit (#28006, Qiagen). The specific antibodies used for these experiments are listed in **Supplementary Table 4**.

### ChIP-qPCR, ChIP-AS-qPCR, and ChIP-seq

For ChIP-qPCR, qPCR was performed at the SNP site in triplicates. The enrichment of TFs at target DNA fragments was quantified relative to IgG controls. Before ChIP-AS-qPCR, primers for allele-specific amplification of the rs4519489 region were designed, with a product length of 234 bp while rs4519489 in the middle of the fragment. Genomic DNA from prostate cancer cell lines (PC3, DU145, 22Rv1, VCaP, and LNCaP) was used as a template for PCR, with Sanger sequencing determining the genotypes at rs4519489. The sequences of oligonucleotides used are listed in **Supplementary Table 2**.

ChIP-seq libraries were prepared using the NEBNext Ultra II DNA Library Prep Kit (#E7103L, NEB) according to the manufacturer’s instructions. Sequencing was performed at Annoroad company. The histone modification (H3K27ac, H3K4me1, and H3K4me3) ChIP-seq libraries were sequenced to yield 150 bp pair-end reads. FastQC (v.0.11.9) was for quality assessment of raw data. Adapters and short reads were removed using TrimGalore (v.0.6.7, RRID: SCR_011847). The trimmed reads were mapped into the human genome Hg38 using Bowtie2 (v.2.2.5)^66^ with the default parameters. Low-quality alignment reads were excluded via SAMtools (v.1.13)^62^ via applying the parameters “-q 30 -F 3844.” Duplicate reads were identified and removed using the Picard toolkit (v.2.25.1, RRID: SCR_006525). MACS2 (v.2.1.4)^67^ was employed for peak calling with default parameters. We utilized the Integrated Genome Viewer (IGV, v.2.12.3) for peak visualization and analysis.

### Expression quantitative trait loci (eQTL) analysis

To evaluate the associations between genotypes of rs4519489 and NOL10 expression levels, we performed an eQTL analysis using the R package “Matrix eQTL” (v.2.2) in the CPGEA cohort comprised of 134 normal prostate samples. The eQTL analysis was applied by fitting a linear regression model (“useModel = modelLINEAR”) between the expression and genotype data, setting up other parameters as default (pvOutputThreshold = 0.05, errorCovariance = numeric ()”). The transcriptional profiling in CPGEA cohort was assessed by RNA-Seq and the CPGEA cohort was genotyped using whole genome sequencing (WGS) strategy.

### EMT score and AR signaling score

The EMT score was based on a set of 76 genes^68^, from which the EMT signature was found correlated with known EMT markers. The AR signaling score was estimated using a gene expression signature from 30 genes^42^, including MPHOSPH9, ADAM7, FOLH1, CD200, FKBP5, GLRA2, NDRG1, CAMKK2, MAN1A1, MED28, ELL2, ACSL3, PMEPA1, GNMT, ABCC4, HERC3, PIP4K2B, KLK3, EAF2, CENPN, MAPRE2, NKX3–1, KLK2, AR, TNK1, MAF, C1ORF116, TMPRSS2, TBC1D9B, and ZBTB10, that was chosen based on their robust activation or inhibition upon androgen stimulation.

### NOL10 cell cycle signature (CCS) and cell cycle progression (CCP) score

The NOL10 cell cycle signature, composed of 32 genes as previously described^9^, was derived from the four top enriched cell cycle related pathways identified via GSEA. The genes from these enriched pathways were then intersected with the 267 genes that were found to be downregulated in our RNA-seq data upon NOL10 knockdown. The CCP score was calculated using a predefined set of 31 CCP genes^69^.

### Multivariate analysis

We investigated the association of the prostate cancer patient biochemical recurrence and overall survival with the NOL10 cell cycle signature and clinical variables, including age, tumor stage, Gleason score, PSA level, seminal vesical status, surgical margin status, and extraprostatic extension status. These factors are critical in understanding the progression and prognosis of prostate cancer. The Cox proportional hazard model was applied to investigate the relation between patient prognosis and NOL10 cell cycle signature. Based on the NOL10 cell cycle signature, samples were stratified into two groups – those with higher expression and those with lower expression. The criterion for stratification was the mean value of the NOL10 cell cycle signature.

### Univariate analysis

For the univariate analysis, we investigated the association of the prostate cancer patients’ biochemical recurrence and metastasis with single or pairwise combinations of gene expression levels of NOL10 and USF1. The z-score sum of gene expression was calculated and patients with prostate cancer were then stratified into two groups – these with higher expression and these with lower expression. The median value of these cumulative expression levels served as the threshold for stratification. Statistics were summarized and presented in forest plots.

### Gene expression correlation analysis

We performed the co-expression analysis to evaluate the expression correlation between NOL10, USF1, NOL10 CCS, CCP, or EMT score from multiple independent cohorts with cancerous prostate tissues. Both Pearson’s product-moment correlation and Spearman’s rank correlation rho methods were applied in all linear expression correlation tests.

### Receiver Operating Characteristic (ROC) analysis

To evaluate the predictive potential functions of the expressions of NOL10 and USF1 for 1-year, 3-year, 5-year, 10-year survival of prostate cancer patients in multiple cohorts, ROC analyses were performed by adding the expression data that were statistically associated with survival to a multivariable adjusted logistic regression model^70^.

### Survival analysis

The Kaplan-Meier survival analysis was conducted to evaluate the impact of SNP genotype or expression levels of NOL10, USF1, or NOL10 CCS on patient prognosis in multiple independent clinical prostate cancer data sets. Patients were stratified based on the SNP genotype or the median value of gene expression levels. For the investigation of the synergistic effect of NOL10 and USF1 on patient survival, we included prostate cancer patients with consensus dual high or low expression levels of NOL10 and USF1. Kaplan-Meier survival analysis was conducted using R package “Survival” (v.3.2.13) and assessed by using the log-rank tests.

### Statistical analysis and data visualization

Throughout the study, continuous variables are presented using the median and interquartile ranges. Discrete variables are reported as the actual number or percentages. All statistical analyses were performed using RStudio (v.1.2.5033) with R environment (v.3.6.3) or unless specified. To determine the expression of NOL10, USF1, or NOL10 CCS on human samples, we compared their expression among normal prostate tissue, primary prostate tumor, and tumor metastasis in multiple prostate cancer clinical cohorts. We evaluated the association of candidate gene expression with other clinicopathological features such as clinical T stages, lymph node metastasis, Gleason score, prostate specific antigen (PSA) level, seminal vesical, person neoplasm status, and BCR. The Mann Whitney U test was used for gene expression in clinical cohorts with two groups, while the Kruskal Wallis H test was applied for cohorts having three or more groups. For the experimental part, data were presented as means ± SD using the GraphPad Prism 6 software. Differences between two groups were estimated using the two tailed student’s T test. The variables in three or more groups were compared using the two-way ANOVA test. Asterisks indicate the significance levels (*P < 0.05; **P < 0.01; ***P < 0.001; ****P < 0.0001). For comparative analyses, P < 0.05 was considered statistically significant. The software and algorithms were listed in **Supplementary Table 5**.

## Data Availability

All data and software used in this study are accessible. The publicly available GWAS data in prostate cancer used in this study were obtained from the GWAS catalog. The publicly available RNA-seq or microarray data including CPGEA, TCGA, MSKCC, GTEx, DKFZ, FHCRC, NPC, Rld, SMMU, Stockholm, SU2C-PCF, Yu (GSE6919), Chandran (GSE10645), Taylor (GSE21034), Grasso (GSE35988), and Penney (GSE62872) were retrieved from public databases including cBioPortal for Cancer Genomics, Oncomine database, and GEO database. The remaining data supporting the findings of this study are available in the Article, Supplementary Information or Source Data file. A reporting summary for this article is available as a Supplementary file. Original data related to the Figures were provided in the Source data files. The deposited data were listed in **Supplementary Table 6**.
